# Potential Association of Molar-Incisor Hypomineralization (MIH) with Dental Agenesis and Infraoccluded Deciduous Molars: Is MIH Related to Dental Anomaly Pattern (DAP)? An Observational Cross-Sectional Study

**DOI:** 10.3390/jcm13082445

**Published:** 2024-04-22

**Authors:** Maria Marcianes, Pablo Garcia-Camba, Alberto Albaladejo, Margarita Varela Morales

**Affiliations:** 1 Unit of Orthodontist, University Hospital Fundación Jiménez Diaz, 28040 Madrid, Spainmemoriavarela@gmail.com (M.V.M.); 2 Department of Surgery, Faculty of Medicine, University of Salamanca, 37008 Salamanca, Spain

**Keywords:** MIH (molar-incisor hypomineralization), agenesis, hypodontia, infraocclusion, deciduous molars, DAP (Dental Anomaly Pattern)

## Abstract

**Background**: Dental Anomaly Pattern (DAP) is a collection of morphologic, numeric, and eruptive anomalies of teeth that are often observed together, suggesting a potential genetic relationship. Our objective was to assess the potential associations of Molar-Incisor Hypomineralization (MIH), a common developmental defect of enamel mineralization with a controversial etiology, with two specific components of DAP: (1) agenesis (AG) and (2) infraoccluded deciduous molars (IODM). Establishing such an association between MIH and one or both anomalies would provide evidence supporting a genetic link between MIH and DAP. **Methods**: We examined pretreatment intraoral standardized photographies and panoramic radiographs from 574 children aged 8–14 years, 287 having MIH and 287 without MIH, comparing the frequencies of AG and IODM in both groups. The subject samples were sourced from the databases of the orthodontic department at a university hospital. **Results:** The frequencies of AG in the MIH and non-MIH groups were 7% and 8%, respectively (*p* = 0.751). The corresponding frequencies of IODM were 27% and 19.2%, respectively (*p* = 0.082). That is, children with MIH did not exhibit an increased frequency of AG or IODM compared to those without MIH. **Conclusions:** These findings do not support the inclusion of MIH in DAP. Nevertheless, further analysis of possible associations is necessary to definitively validate or invalidate this hypothesis.

## 1. Introduction

**Figure 1:**
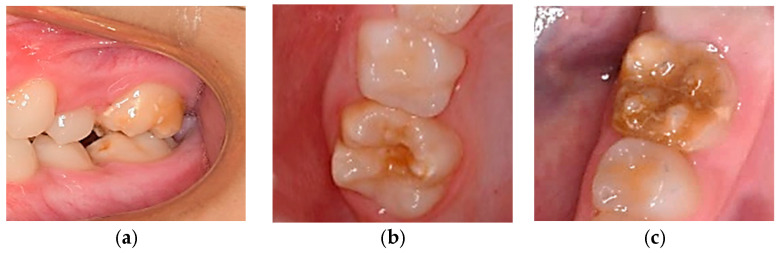
(**a**–**c**): MIH: Mild, moderate, and severe involvement of the first permanent molars.

**Figure 2:**
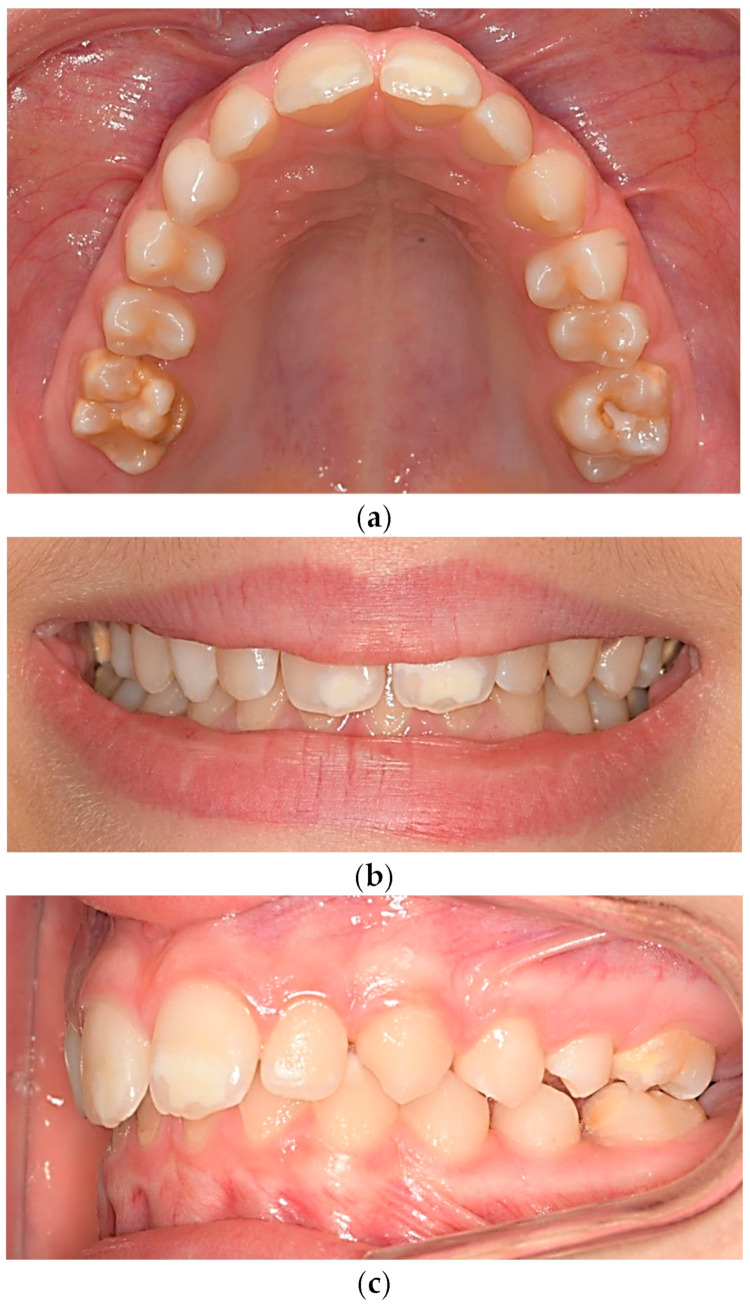
(**a**–**c**): MIH: Involvement of central incisors with diverse degrees of expression of hypomineralization.

Molar-incisor hypomineralization (MIH) is a developmental defect in enamel mineralization that affects one or more first permanent molars (FPM), occasionally involving permanent incisors [1]. Clinically, MIH manifests as asymmetrical demarcated opacities with variable color, extent, and severity, primarily on the cusps and incisal third of affected molars (Figure 1). In cases involving incisors, opacities appear on the vestibular surfaces (Figure 2).

MIH may result in thermal hypersensitivity, post-eruptive breakdown, caries, and adverse aesthetic and psychological effects [2], necessitating early prophylactic measures and interdisciplinary management from treatment design to completion [3]. The histopathology of enamel in MIH exhibits reduced mineral concentration, while preserving ameloblast function and maintaining normal enamel thickness [4].

Reported MIH prevalence varies widely, ranging from 2.8% to a peak of 40.2% in one epidemiological study [5]. More recent research records a maximum frequency below 30%, attributing the broad range to methodological variations among studies, with a lack of uniform diagnostic criteria [6]. Consensus on diagnostic criteria was only achieved in 2003 at the Congress of the European Academy of Paediatric Dentistry (EAPD) [7] and later updated. Nevertheless, the use of non-standardized criteria persisted among some authors, hindering systematic reviews and meta-analyses [6]. A 2021 systematic review by Lopes et al. reported a pooled MIH prevalence of 13.5%, with no significant differences across continents [8]. That review included 116 observational studies published between 2003 and 2021, with the majority applying the diagnostic criteria of the EAPD. Most of the MIH lesions are mild, with only about 7% being classified as severe [9]. In the meta-analysis by Lopes et al., the proportion of cases classified as moderate to severe was 36% [8]. It has been reported that an average of 878 million people suffer from MIH worldwide and that 17.5 million new cases appear each year [10]. The high worldwide prevalence of MIH, along with its effects on the quality of life for many patients and the economic impact of treatments, has led to the recognition of this condition as a potential public health issue [11].

The initially idiopathic nature of MIH has sparked ongoing debate on its etiology. Recent research supports MIH as a likely multifactorial genetic condition, involving mutations in multiple genes related to odontogenesis, along with environmental influences during prenatal, perinatal, and postnatal periods [12,13,14,15]. Considering this genetic background, studying associations with disorders occurring more frequently than chance can provide indirect evidence of shared genetic bases. In 1998, Baccetti [16] identified reciprocal associations among some dental anomalies, including dental agenesis (AG) and infraocclusion of deciduous molars (IODM), with an enamel mineralization disorder that he named ‘hypoplasia’. He defined hypoplasia as “a break in continuity or surface loss of enamel that is not related to dental caries or trauma”. This definition does not specify whether enamel loss was exclusively pre-eruptive, a distinctive characteristic of genuine hypoplasia, or post-eruptive, which is typical of MIH. Given the much higher prevalence of MIH compared to genuine hypoplasia, Baccetti’s sample likely included MIH cases alongside fewer genuine hypoplasias.

### Dental Anomaly Pattern (DAP)

In an editorial published in 2009, Sheldon Peck consolidated the concept of Dental Anomaly Pattern (DAP) based on previous studies about associated dental anomalies [17]. He described DAP as the finding of “associated heterogeneous dental abnormalities involving the morphology, number and eruption of teeth that are observed together much more frequently than can be explained by chance alone and appear to have a common genetic cause”. Peck’s original list comprised 9 anomalies (Table 1), co-occurring in various combinations of two or more.

The enamel mineralization disorder previously described as enamel hypoplasia by Baccetti [16] was not initially a component of DAP. However, Peck left room for new additions, if any anomaly added would share a genetic relationship with those initially listed. Since then, associations with other disorders, such as taurodontism and dental transpositions [18,19], and also MIH [20,21,22], have been explored, yielding conflicting results.

The purpose of this study was to analyze potential associations between MIH and two anomalies in Peck’s DAP list: (1) agenesis (AG) and (2) infraocclusion of deciduous molars (IODM). Confirming these associations would support a genetic link between MIH and these anomalies and the potential inclusion of MIH in the list of DAP. The null hypothesis tested in this study was as follows: there is no statistically significant association between the presence of AG, IODM or both anomalies and the occurrence of MIH in the study population.

## 2. Materials and Methods

### 2.1. Study Design

This observational cross-sectional study was conducted as part of a comprehensive research project at the Orthodontics Unit of the University Hospital Fundación Jiménez Díaz (UHFJD) in Madrid, Spain (EO131-19_FJD). The project comprised two branches: the first aimed to explore the potential relationships between MIH and hypomineralized second primary molars (HSPM), focusing on the predictive value of HSPM for the subsequent development of MIH. The findings from this initial study have been previously reported [23]. In the current branch of the investigation, the objective was to explore the possible association of MIH with two dental anomalies, AG and IODM, which are components of DAP.

### 2.2. Study Population and Procedure

A sample of 560 children was selected, comprising 287 patients with MIH (160 female and 127 male) and 287 age-matched patients without MIH (155 females and 132 males). The sample sizes were determined through a previous pilot study conducted with 50 patients with MIH and 50 without MIH. The sample size calculation was based on a significance level (α) of 0.05 and a power of 0.80. The prevalence of hypodontia in the control group was estimated at 7%, with a moderate effect size (Cohen’s d = 0.5). Thus, a total sample size of 574 participants (287 per group) was determined to detect differences in hypodontia frequency between patients with inciso-molar hypomineralization (MIH) and the control group without MIH. With respect the frequencies of IODM, considering its prevalence to be approximately 25%, a sample size of 382 participants (191 in the MIH group and 191 in the non-MIH group) would be sufficient to detect a moderate effect size in the frequencies between the two groups.

Both samples were obtained at the Orthodontic Unit of UHFJD, Madrid, Spain. All subjects had been entered into the database of the Unit prior to the start of any orthodontic treatment. The children’s age ranged from 8 to 14 years when their first diagnostic records were obtained. The selection of subjects for the MIH sample began with the last patient included in the afore mentioned database who met the EAPD diagnostic criteria for MIH [7], as shown in Table 2. Subsequent patient recruitment proceeded in reverse order. The non-MIH comparison sample of subjects was assembled in the same way.

### 2.3. Evaluation of MIH Lesions 

MIH lesions were evaluated using high-quality digital intraoral photographs displayed on a 40-inch screen [24]. Following the criteria outlined by the EAPD, a diagnosis of MIH was established if at least one FPM exhibited hypomineralization. The remaining FPMs could be unaffected, or even absent. Therefore, the presence of all FPMs was not mandatory for inclusion in the MIH sample when at least one molar was affected by MIH. Incisor lesions were categorized as MIH only if at least one FPM showed involvement. For this study’s scope, consensus was reached that the most extensive MIH opacity must have a minimum diameter of 1 mm, aligning with the Developmental Defects of Enamel (DDE) index [25]. To prevent false-negative cases for MIH, subjects in the non-MIH sample needed to have all four FPMs present and free of the disorder. Cases with an unclear diagnosis of MIH based on the EAPD criteria were excluded. For instance, patients with cavities that were not easily distinguishable as typical of MIH, as opposed to ‘conventional’.

### 2.4. Inclusion/Exclusion Criteria

Table 3 outlines the inclusion/exclusion criteria applied in the selection of the samples.

Most panoramic radiographs (90%) had been taken with an ORTOPHOS model from SIRONA (Sirona Dental Systems GmbH; Bensheim, Germany). The device has automatic selection of the planographic shape by adjusting temple support for different maxillary arches. The frame format was 15 × 30 and a photostimulable phosphor plate was used as the image acquisition device. The remaining radiographs obtained with other devices exhibited a similar high quality.

### 2.5. Training and Calibration of the Examiners

The selection of MIH and non-MIH patients was performed by 2 examiners (M.M and P.G.C) previously trained and calibrated by a senior researcher considered the gold standard (M.V), all co-authors of the research. A set of 50 photographs, which included images of MIH along with other enamel defects such as fluorosis, white spots, hypoplasia, amelogenesis imperfecta, and other opacities and discolorations, was used for training and calibration. The Cohen’s Kappa inter-examiner coefficient was rated as outstanding (0.85) and intra-examiner reliability tests revealed near-perfect agreement. Training and calibration for AG and primary IODM diagnosis were conducted using a set of 100 high-quality panoramic radiographs. Full inter-observer agreement was found when analyzing images for both AG and IODM. 

### 2.6. Association of MIH with AG and Primary IODM

In both the MIH and non-MIH groups, we separately calculated the frequencies of AG and IODM.

*Agenesis*

Dental AG is characterized by the congenital absence of a variable number of teeth due to the lack of formation of the corresponding tooth germ. In this study, dental AG was determined through panoramic radiography (OPG) screening for absent permanent teeth, excluding third molars. We verified past dental histories to ensure that the absence of permanent teeth resulted from primary germ development failure rather than extraction or other acquired causes. To rule out late germ development, a second OPG was obtained after the age of 12 for all subjects under 10, as explained in the inclusion/exclusion criteria section. To calculate the frequency of AG, we used complete samples of 287 patients each since it was not necessary to exclude any subjects. 

*Infraocclusion of deciduous molars*

IODM refers to a deciduous molar that undergoes a gradual loss of contact with opposing teeth and assumes an inferior position to the occlusal plane. It can be primary, possibly due to genetic factors or secondary, resulting from acquired causes. In our study, we investigated the association between MIH and primary IODM. Patients with secondary IODM or ankylosis due to trauma, inflammatory lesions with coronary destruction, large interproximal fillings, stainless steel crowns, or pulp treatments were not considered positive for IODM.

**Figure 3:**
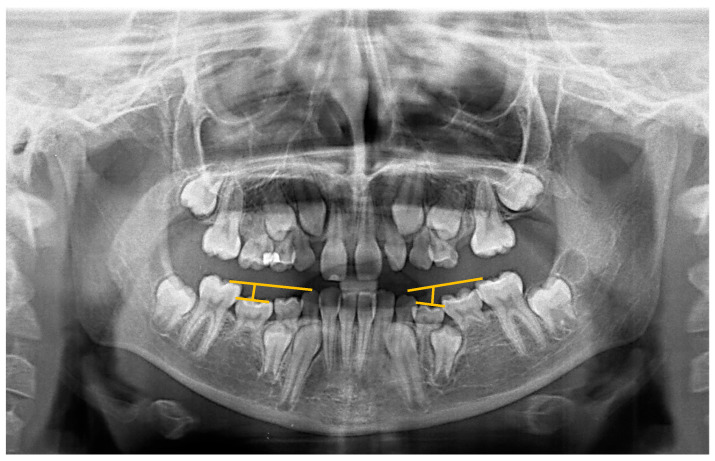
Odeh’s method for diagnosing IODM on a panoramic radiograph. This method is based on three lines. First line: from the medial marginal ridge of the FPM to the cusp tip of the homolateral primary canine. Second line: from the distal to the medial marginal ridge of the primary molar. Third line: a perpendicular from the mid-point of the second line to the occlusal plane. This distance is measured in millimeters.

IODM was assessed on panoramic radiographs (OPG) using the method by Odeh et al. [26] (Figure 3). This method involves three lines, where the first extends from the medial marginal ridge of the first primary molar to the cusp tip of the corresponding primary canine, representing the occlusal plane. For patients lacking the homolateral deciduous canine, the method by Bjerkin et al. [27], modified by Cardoso et al. [28], was used. With this method, the IODM magnitude is measured on a perpendicular extending from a line that connects the medial and distal marginal ridges to the occlusal plane. IODM was considered present when the discrepancy exceeded 1 mm using both methods. While some authors, like Shalish et al. [29], use models for IODM measurement, Odeh et al. found that measurements on radiographs were comparable to those using models [26].

To analyze the frequency of IODM in MIH and non-MIH patients, we required that subjects have all deciduous molars preserved unless one or more were infraoccluded. In that case the subject was considered positive for IODM. This criterion resulted in the exclusion of some subjects including almost all older subjects, most of whom did not retain all their deciduous molars. However, a small number of 13- and 14-year-old children with delayed dental eruption met the afore mentioned criteria. They preserved all their second deciduous molars or lacked one or more, but at least one exhibited infraocclusion; therefore, they were not excluded. As both samples were age-matched, the number of subjects excluded from each was comparable. The final sample sizes to analyze frequencies of IODM were MIH, *n* = 204; non-MIH, *n* = 203.

### 2.7. Statistical Analysis

The mean and standard deviation were determined for the age variable. For qualitative variables, such as AG and IODM, absolute and relative frequencies were applied. Student’s *t*-test was used to compare the mean patient age, and the Chi-square test was used to compare percentages. The level of significance was set at *p* < 0.05.

### 2.8. Ethical Approval

The clinical research was approved by the Ethics Committee for Research in Humans of University Hospital Fundación Jiménez Díaz, under procedure number EO131-19_FJD. All methods were in accordance with approved guidelines. Informed consent from the parents or guardians of all children included in this study was available.

## 3. Results

The frequency of MIH in the population from which the samples were drawn, within the same age range, was 12.8%. The frequency of MIH in the orthodontic population of the same age, from which the samples were obtained, was 12.64%, with a male-to-female ratio of 1.26:1. 

### 3.1. Demographic and MIH Characteristics

Table 4 displays the age and gender distribution of subjects included in the MIH and non-MIH samples at the time the original samples were compiled. No significant differences were observed in any case. 

Among the 287 subjects in the MIH sample, 48% exhibited exclusive molar involvement, while 52% showed involvement of both, molars and incisors. Severity distribution was as follows: 26% severe cases, 35% mild cases, and 38% moderate cases. No significant differences were observed regarding the involvement of 1, 2, 3, or 4 FPMs. A consistent relationship was observed between the number of affected FPMs and the severity of MIH. MIH was severe in only 6.5% of children with a single affected molar, rising to 48% for those with all 4 molars affected (*p* < 0.001).

### 3.2. Frequencies of AG in Patients with and without MIH

Table 5 and Table 6 display the respective frequencies of AG of any teeth (excluding third molars) and AG of premolars for the samples of patients with and without MIH. The differences were not significant in either case.

### 3.3. Frequencies of Primary IODM in Patients with and without MIH

Table 7 shows the respective frequencies of primary IODM in the reduced samples of patients with and without MIH. No significant differences were observed.

## 4. Discussion

This study is part of comprehensive research on the associations between MIH and other dental anomalies. We had previously investigated the predictive value of hypomineralization of second primary molars (HSPM) for MIH, with a clinical emphasis on preventive measures when detecting HSPM in young children. Its results confirmed our hypothesis, demonstrating that the presence of hypomineralized second primary molars (HSPM) in the primary dentition predicts the subsequent development of MIH in permanent teeth [23]. The current branch of the research operates on a distinctly different foundation, seeking potential associations between MIH and other anomalies that share a certain genetic basis. The objective was to refine our understanding of MIH etiology.

When formulating this study, we aimed to minimize, as much as possible, certain methodological limitations and inconsistencies identified in published studies on MIH, as highlighted by Crombie et al. [30]. These authors specifically mentioned issues such as the lack of diagnostic accuracy for MIH and advocated for enhancing the standardization of procedures. 

The present study began by training evaluators to assure their proficiency in recognizing the various presentations of MIH. This training is crucial to prevent potential confusion with other conditions that need distinction from MIH. These conditions include fluorosis, enamel hypoplasia, amelogenesis imperfecta, white spot lesions, and enamel discolorations [31]. We adhered to MIH diagnostic criteria established by the EAPD in 2003 [7], which were further discussed during the Seminar and Workshop on MIH held in Helsinki in 2009 [5]. The consensus reached during this event resulted in the publication of the “Best Clinical Practice Guidance for clinicians dealing with children presenting with Molar-Incisor-Hypomineralisation (MIH)” [32]. This document included a treatment decision-making guide, along with suggestions on etiology and recommendations for future research and was updated in 2022 [3]. The release of the EAPD criteria marked a significant milestone in epidemiological and clinical research on MIH, providing a framework to delineate this defect nosologically and diagnostically.

To train evaluators and validate the clinical diagnosis of MIH recorded in the database, we scrutinized high-quality photographs on a 40-inch screen. Previous research has established the suitability of intraoral photographs for diagnosing MIH [24]. Diagnosis training for AG and IODM was conducted using panoramic radiographs, resulting in complete agreement among evaluators. This aligns with expectations, as these pathologies are readily diagnosable through clinical and/or radiographic means.

The age range of patients at the initiation of this study was between 8 and 14 years. The optimal age for diagnosing MIH is approximately 8 years when the four FPMs are typically fully erupted in the majority of children and the likelihood of complications masking the defect is low [33,34]. However, considering the objective of our study, we set the upper age limit at 14 years, a period when the chances of dental mutilations or losses due to causes other than MIH remain low. The 8–14 years range is suitable for analyzing the presence of AG, as some cases cannot be confirmed in very young children due to the possibility of late tooth-germ development [35]. Consequently, in this study, a second radiograph obtained after the age of 12 years was required for the diagnosis of AG in children under 10 years of age. Beyond the age of 14, the diagnosis of AG (excluding third molars) can be almost irrefutable, provided that acquired causes for the absent teeth have been ruled out. On the other hand, the upper limit of 14 years might appear somewhat high for evaluating IODM because, by the age of 12, many children no longer retain any deciduous molars. However, deciduous molars with infraocclusion tend to exfoliate later [36]. Considering that to diagnose IODM, it was enough to have a single deciduous molar with the anomaly, extending the upper age limit to 14 years seemed acceptable. Other researchers studying IODM have also set upper limits beyond 12 years [22,37]. The survival of second deciduous molars, including those with infraocclusion, is particularly long in cases with agenesis of premolar successors, as demonstrated in a systematic review [38]. All studies reported an average survival rate ranging from 82% to 89% for second deciduous molars in the oral cavity after 5 to 13 years of follow-up.

The aim of this study was to explore the potential relationship between MIH and two disorders exhibiting associative patterns that may have a genetic link: AG and IODM. Therefore, the analysis focused on the frequency of primary IODM, with no apparent acquired cause. Cases involving ankylosis due to local traumatic injury or inflammation of Hertwig’s epithelial root sheath, which are the most frequent causes of secondary infraocclusion [39], were not considered. Although clinically similar, primary and secondary IODM are entirely different pathophysiologically, the frequency of AG in subjects with and without MIH was measured in complete samples, each comprising 287 subjects. However, for the analysis of IODM frequency, it was necessary to exclude subjects from both samples in whom the determination of the presence or absence of IODM could not be ascertained. 

Regarding the presence of deciduous molars in the original MIH and non-MIH samples, three potential scenarios were identified: Some subjects retained all deciduous molars, allowing for the confident determination of the presence or absence of IODM, thus enabling the categorization of subjects as positive or negative for IODM. Therefore, these patients were included in the samples. Other subjects lacked one or more deciduous molars, but at least one of those remaining exhibited IODM. These subjects should be considered IODM positive, regardless of the absence of other deciduous molars, and they could also be included in the analysis. Finally, some subjects lacked one or more deciduous molars, and those present did not exhibit IODM. In such cases, it was impossible to determine whether the missing deciduous molars presented infraocclusion before exfoliation. Consequently, these cases were deemed invalid for the IODM frequency analysis and were excluded from the samples. This exclusion resulted in a reduction in the sample sizes for both the MIH and no-MIH groups from 287 patients each to 204 and 203, respectively. Among the patients excluded were many children aged 12 years and older, as they were more likely to have lost one or more deciduous molars. The strict age matching of the MIH and no-MIH samples ensured a similar number of excluded patients in both groups, minimizing potential age-related bias. This consideration, overlooked by other authors [38], highlights that some subjects counted as negative for the anomaly in their studies might have been positive if any of the absent deciduous molars had exhibited IODM.

Among all anomalies that frequently form associative patterns (DAP) with other disorders listed by Peck [17], we chose to separately analyze AG and primary IODM for their potential association with MIH. This decision was based on the ease of establishing a precise and reproducible diagnosis in both cases. Additionally, these anomalies are involved in various established associations [29,40]. 

While some authors rely on simple clinical observation for diagnosing IODM, we employed two objectives based on measurements using panoramic radiographs. In most subjects, we utilized Odeh’s method, with one of its reference points being the cusp of the homolateral deciduous canine. In cases where no such deciduous canine was present, we employed the method of Bjerkin et al. [27], as modified by Cardoso et al. [28], which does not require this specific point of reference. Odeh et al. found that measurements taken from radiographs did not significantly differ from those performed in models [26]. Furthermore, the threshold established for diagnosing IODM (i.e., a 1 mm discrepancy) remains consistent across all methods.

As stated previously, AG and primary IODM are components of various established associations. Numerous studies have demonstrated associations between AG and transpositions [41], microdontia [42], impacted maxillary canines [43], and delayed eruption of teeth [44]. On the other hand, primary IODM has been linked to microdontia of the maxillary lateral incisors, maxillary canine impaction, and distoangular deviation of the second lower premolar [29]. While IODM has also been associated with AG [40], our study did not aim to analyze this association. Instead, our objective was to separately compare the frequencies of AG and primary IODM in subjects with and without MIH.

In this study, the frequencies of both AG and primary IODM were comparable among patients with and without MIH, which would rule out a potential association between MIH and either of these disorders. Additionally, in line with Peck’s hypothesis, it neither suggests a genetic etiological link nor supports the inclusion of MIH in the list of anomalies forming associative patterns (DAP).

There are few studies in the literature that analyze the potential relationship between MIH or other congenital enamel mineralization defects and dental anomalies that may manifest clinically through associative patterns (DAP), such as AG and IODM. In a significant research study predating Peck’s editorial on DAP, Baccetti [16] identified a noteworthy association between a disorder of enamel mineralization, which he referred to as hypoplasia, and several dental anomalies, including AG and IODM. The term ‘hypoplasia’ has frequently been inaccurately used to describe both qualitative and quantitative enamel defects. A notable limitation of the DDE index in scoring MIH is its tendency to misclassify post-eruptive enamel loss as hypoplasia [45]. Hypoplasia, a pre-eruptive quantitative enamel anomaly associated with decreased thickness, becomes apparent upon tooth eruption, presenting as pits, grooves, missing enamel, or smaller teeth. In contrast, MIH is a qualitative defect that can progress with acquired post-eruptive breakdown. Furthermore, the margins of hypoplastic opacities are smooth, while in MIH, they exhibit irregularities [46]. Nonetheless, clinical differentiation between these two anomalies can be challenging.

Baccetti [16] published his article before the establishment of the EAPD criteria for defining MIH. Like other authors of that time, he likely grouped defects of mineralization, including some MIH cases with post-eruptive breakdown, a disorder more prevalent than true hypoplasia [47], under the category of ‘hypoplasias’. A similar situation is evident in the study by Lai and Seow [20], who analyzed enamel hypoplasias in a sample of children with AG and found a significant association between the two anomalies. Similar to the Baccetti study [16], defects considered hypoplasias by Seow may have actually been MIH.

The studies that have analyzed the association of MIH, defined according to the EAPD criteria, and different anomalies including AG and IODM are scarce and have been recently published. In the first study by Walshaw et al. [21], conducted on a sample of 101 patients with MIH sourced from a dental hospital in Great Britain, the reported frequencies of AG and IODM were 12% and 9%, respectively. The most frequent type of AG associated with MIH was AG of the premolars. Based on these frequencies, the authors concluded that MIH was associated with AG and IODM. However, their study lacked a comparison group, a limitation that they themselves acknowledged.

In the present study, we found a similar frequency of AG in patients with MIH as observed by Walshaw et al. [21], and an even higher frequency of IODM in the same patients, aligning with the findings of other authors in our geographical surroundings [48]. This may lend support to Walshaw et al.’s conclusion. However, our study included a comparison sample of patients without MIH, and we did not observe significant differences between the frequencies of AG or IODM between patients with and without MIH. Hence, we cannot endorse Walshaw et al.’s conclusion based on our results. 

Betül Sen Yavuz et al. [22] in a sample of 429 children with MIH and 437 controls without MIH aged 8 to 14 years, analyzed the frequency of 12 anomalies, including AG and IODM. Twelve percent of their subjects exhibited one or more anomalies. The methodology utilized in this research, based on clinical examinations and panoramic radiographs, was robust, with a substantial sample size of 429 subjects. However, anomalies were grouped to ensure statistical power. Their findings aligned with ours, did not show an association between MIH and other dental anomalies. Our sample was smaller, although it surpassed Walshaw et al.’s. Possibly, this fact limited the detection of minor effects due to statistical power. Nonetheless, the analysis still provides valuable insights into observed trends.

Finally, it is worth mentioning a large-scale multicenter protocol currently being developed, involving 22 research groups from 15 countries located on all continents [49]. The objective is to investigate possible associations of MIH with other dental developmental anomalies, in different geographical locations. They aim to recruit 584 subjects with MIH aged between 7 and 17 years and a comparison group with 584 subjects each in pediatric dentistry clinics. Data are obtained from clinical examination and panoramic radiography following a standard diagnostic approach. The research is in preliminary stages, but its results promise to be enlightening.

## 5. Limitations

1. Observational cross-sectional design limits longitudinal analysis.
2. Limited number of patients with AG and/or IODM restricts in-depth data exploration beyond frequency analyses.
3. Exclusively studying an orthodontic population may limit broader applicability.
4. Geographical specificity to Madrid, Spain, may restrict generalizability to populations with different prevalence rates of MIH, AG, and IODM.

## 6. Conclusions

No significant associations were identified between MIH and dental agenesis or infraocclusion of deciduous molars. This finding does not support a genetic link between MIH and these disorders, nor does it indicate that MIH should be included among anomalies forming associative patterns (DAP). However, to conclusively rule out this inclusion, further studies are required to explore potential associations between MIH and other DAP-related disorders, such as maxillary palatal canine impaction or angular positioning of the unerupted mandibular second premolar. 

## Figures and Tables

**Table 1:** Dental Anomalies Co-Occurring More Than Expected by Chance (DAP): Peck’s Original Paper Listing [17].

Absent teeth Microform teeth (eg, peg-shaped lateral incisor) Tooth-size reduction (generalized or localized) Delay in tooth formation and eruption (generalized or localized) Infraocclusion (most often of deciduous teeth) Palatal displacement of canine Maxillary canine-first premolar transposition (Mx.C.P1) Mandibular lateral incisor-canine transposition (Mn.I2.C) Distal angulation of unerupted mandibular second premolar

**Table 2:** EAPD diagnostic criteria for MIH.

At least one FPM affected by the defect * White-yellow-brown demarcated opacities Post-eruptive enamel breakdown (PEB) associated with opacities Extensive atypical caries with surrounding opacities or in low-risk surfaces Atypical restorations; crowns if MIH is found in other teeth Extractions due to MIH Eruption failure of a molar or an incisor

* Lesions on the incisors are only diagnosed as MIH if at least one FPM is also affected.

**Table 3:** Inclusion/exclusion criteria in MIH and no-MIH samples.

Absence of other developmental defects (dentinogenesis imperfecta, amelogenesis imperfecta, fluorosis) Absence of syndromes or craniofacial anomalies Lack of consanguinity with other selected subjects Availability of high-quality panoramic radiographs and digital intraoral photographs onto a 40-inch screen Patients with agenesis who were younger than 10 years were required to have a second OPG obtained after 12 years of age to be included in the study.

**Table 4:** Age and gender of patients with and without MIH.

	MIH	No MIH	*p*
Total	287	287	
Age (years)	9.15 ± 1.92	9.57 ± 1.65	0.005
Gender: F/M	160/127	155/132	0.737

**Table 5:** Frequency of agenesis of any tooth, excluding third molars, in patients with and without MIH.

AG *	MIH	No MIH	*p*
Yes	20 (7%)	23 (8%)	0.751
No	267 (93%)	264 (92%)	
Total	287	287	

* AG: agenesis.

**Table 6:** Frequency of agenesis of premolars in patients with and without MIH.

AG * of Premolars	MIH	No MIH	*p*
Yes	14 (4.9%)	13 (4.5%)	1.000
No	273 (95.1%)	274 (95.5%)	
Total	287	287	

* AG: agenesis.

**Table 7:** Frequency of infraocclusion of deciduous molars in patients with/without MIH.

IODM *	MIH	No MIH	*p*
Yes	55 (27%)	39 (19.2%)	0.082
No	149 (73%)	164 (80.8%)	
Total	204	203	

* IODM: infraocclusion of deciduous molars.

## Data Availability

The data presented in this study are available on request from the corresponding author. The data are not publicly available due to privacy/ethical reasons.
